# High‐density mutation tracks are associated with proton‐beam irradiation patterns in *Sorghum bicolor*

**DOI:** 10.1002/tpg2.70267

**Published:** 2026-06-29

**Authors:** Ezekiel Ahn, Insuck Baek, Seunghyun Lim, Louis K. Prom, Moon S. Kim, Lyndel W. Meinhardt, Clint Magill

**Affiliations:** ^1^ Sustainable Perennial Crops Laboratory, Agricultural Research Service, United States Department of Agriculture Beltsville Maryland USA; ^2^ Environmental Microbial and Food Safety Laboratory, Agricultural Research Service, United States Department of Agriculture Beltsville Maryland USA; ^3^ Insect Control and Cotton Disease Research, Agricultural Research Service, Southern Plains Agricultural Research Center, United States Department of Agriculture College Station Texas USA; ^4^ Department of Plant Pathology and Microbiology Texas A&M University College Station Texas USA

## Abstract

Induced mutagenesis is a cornerstone of crop functional genomics, yet the extent to which distinct radiation sources reshape the spatial distribution of mutations remains difficult to evaluate in reduced‐representation datasets. Here, we analyze a published genotyping‐by‐sequencing (GBS) panel (192,040 loci) to compare proton‐beam and gamma‐ray mutagenesis in *Sorghum bicolor*. Because GBS sampling is nonuniform, all analyses were conducted within an explicitly defined GBS‐callable sequence space. Within this callable space, 96‐channel trinucleotide spectra were broadly similar between radiation types, whereas spatial summaries differed. Macroscale analysis using the Gini coefficient indicated that proton‐treated lines exhibit a highly unequal, spike‐like distribution of mutations, whereas gamma‐treated lines show a more diffuse window‐level distribution. Microscale spatial statistics were consistent with clustering patterns that were more prominent in proton‐treated lines, including an aggregation scale of ∼500 kb, with a substantial fraction of the mutational burden falling into high‐density tracks. Within the callable locus set, coding‐ and promoter‐proximal categories were not depleted of induced mutation events (single‐nucleotide variants) across treatments. Furthermore, we did not detect a negative association between total mutational load and the coding‐region mutation fraction in this dataset. These findings suggest that, within this dataset, proton mutagenesis is characterized not by unique chemical signatures but by a distinct spatial geometry that concentrates detectable mutation events. Because proton irradiation was represented by a single dose, whether proton treatment produces stronger clustering than gamma irradiation at equal mutational burden remains to be directly tested.

## Core Ideas

- Callable‐space correction enables fair comparisons in reduced‐representation mutagenesis datasets.
- Proton‐beam irradiation concentrates mutations into high‐density genomic tracks.
- Gamma irradiation shows a more diffuse mutation landscape within the same callable space.
- Proton and gamma lines share broadly similar trinucleotide mutation spectra.
- Track‐like clustering, not unique chemistry, best distinguishes proton mutagenesis in this dataset.

## Plain Language Summary

Proton beams and gamma rays are used to create mutations for crop breeding, but they may damage DNA in different physical ways. We reanalyzed a published sorghum genotyping‐by‐sequencing (GBS) dataset to compare where induced mutations fall along the genome after proton‐beam versus gamma‐ray irradiation. Because GBS samples the genome unevenly, we defined the measurable “callable space” for each line and restricted all comparisons to that space. Within callable space, the two radiation types showed similar base‐change patterns, but proton‐treated lines had a far more uneven, spike‐like mutation landscape. Spatial analyses showed stronger clustering in proton lines, with many mutations concentrated into high‐density tracks on the scale of hundreds of kilobases. These results suggest that, in this dataset, proton mutagenesis is distinguished mainly by where detectable mutation events occur, highlighting why callable‐space correction is essential for reduced‐representation mutagenesis studies.

**Abbreviations**
CDS: coding sequence
GBS: genotyping‐by‐sequencing
LET: linear energy transfer
MBS: multi‐base substitution
PCA: principal component analysis
SNP: single nucleotide polymorphism
SNV: single‐nucleotide variant
TE: transposable element
Ts/Tv: transition/transversion

## 1 INTRODUCTION

Induced mutagenesis is a cornerstone of crop functional genomics and breeding, enabling the rapid expansion of allelic diversity without the introduction of foreign DNA (Oladosu et al., 2016). While gamma irradiation has long been the workhorse of mutation breeding due to its scalability and predictable dose‐response, the increasing accessibility of particle irradiation, specifically proton beams, raises a critical question for modern plant genomics: Do distinct radiation sources merely alter the quantity of mutations, or are differences primarily evident in the spatial distribution of variants within the accessible sequence space?

From a biophysical perspective, this distinction is pivotal. Gamma rays are low‐linear energy transfer (LET) radiation, depositing energy sparsely and randomly (Nikjoo et al., 2016). In contrast, charged particles such as protons can deposit energy along defined physical tracks, with higher local ionization density than sparsely ionizing gamma rays, potentially generating clustered DNA lesions (Goodhead, 1994). In mammalian systems, such particle tracks have been linked to complex double‐strand breaks that challenge cellular repair mechanisms (Goodhead, 1994; Nikjoo et al., 2016; Sage & Harrison, 2011). However, in complex plant genomes, characterized by high compartmentalization into gene‐rich euchromatin and repeat‐dense heterochromatin, the “geometry” of these lesions remains largely unexplored (Jo & Kim, 2019).

This genomic architecture adds an evolutionary dimension to the biophysical problem. If high‐LET radiation concentrates damage into specific genomic domains, it may disproportionately impact functional genes versus repetitive elements. Furthermore, extreme biophysical stress may trigger systemic genome instability, resonating with concepts of stress‐induced genome instability (McClintock, 1984). While direct structural rearrangement is difficult to detect with reduced‐representation sequencing, the spatial distribution of mutations can serve as a proxy for understanding how the genome accumulates damage under such acute physical stress (Fedoroff, 2012).

In this study, we provide a comprehensive physical genomic analysis of proton‐beam versus gamma‐ray mutagenesis in *Sorghum bicolor*. Leveraging a large‐scale dataset of putative induced single‐nucleotide variant (SNV) events derived from 96 sorghum lines (Lee et al., 2023), we move beyond simple mutation counting to quantify the biophysical geometry of damage. We integrate multi‐scale spatial statistics, from macroscale chromosomal landscapes to discrete mutation track calling, to reveal the structure of proton‐induced lesions. Furthermore, we connect this spatial geometry to the biological context by assessing callable‐space feature enrichment, multi‐base substitution (MBS) rates, and the relationship between mutational load and coding‐region mutation fraction (Kimura, 1985; Nei, 2005). Together, these analyses suggest that proton‐ and gamma‐treated lines differ more strongly in the spatial geometry of induced SNV events than in trinucleotide spectra, motivating the use of explicit callable‐space definitions for reduced‐representation mutagenesis studies.

## 2 MATERIALS AND METHODS

### 2.1 Plant materials and data acquisition

This study utilized a genomic and phenotypic dataset derived from a diverse panel of 96 *S. bicolor* genotypes, comprising 37 natural germplasm accessions and 59 mutagenized lines generated via gamma‐ray (100–400 Gy) and proton‐beam (300 Gy; n = 9) treatments, as previously described by Lee et al. (2023). All primary biological materials, irradiation procedures, and agronomic trait measurements were performed in the source study.

### 2.2 Genotypic data processing and induced mutation calling

Genotype calls were retrieved from the previously published genotyping‐by‐sequencing (GBS) dataset containing 192,040 high‐quality single nucleotide polymorphisms (SNPs) for the 96 lines (Lee et al., 2023). To identify induced mutations specific to this study's biophysical analysis, we performed a pairwise mutant–parent comparative analysis. Unlike standard population genetics approaches, we strictly compared each mutant line against its specific biological parent (Origin). Variants present in the mutant and represented as reference calls in the matched parent were operationally classified as induced SNV events. This operational definition reduces contributions from standing variation present in the parental line, while acknowledging that residual heterozygosity and genotype‐call uncertainty can contribute to false positives. Induced SNV events were evaluated only at loci present in the published, filtered GBS SNP panel, rather than from genome‐wide de novo variant discovery. Because the analysis was based on the published filtered genotype matrix rather than raw GBS read alignments, per‐site read depth, allele balance, and genotype‐quality thresholds could not be reestimated in this study. Consequently, these calls should be interpreted as putative mutant–parent genotype differences within the filtered GBS panel, and residual false positives from allele dropout, residual heterozygosity, or genotype misclassification cannot be excluded.

### 2.3 Callable‐space framework and GBS‐accessible denominators

Because GBS provides nonuniform genomic sampling, all rate and enrichment analyses were performed within the GBS‐accessible callable space rather than the full genome. The callable space was defined from the published SNP matrix provided by the source study (Lee et al., 2023) across chromosomes 1–10. For each line, a locus was considered callable if it contained a valid genotype call in the matrix, including unambiguous bases (A/C/G/T) and International Union of Pure and Applied Chemistry ambiguity codes representing ambiguous/heterozygous calls (R/Y/S/W/K/M), whereas N/n/empty calls were treated as missing. Sample‐level callable loci counts and missingness were summarized by radiation group (Figure S1; Supporting Information Data S1).

To obtain feature‐specific callable denominators, callable loci were annotated against the Rio v2.1 gene models (coding sequence [CDS], promoter, gene body non‐CDS, and intergenic) and repeat‐masked intervals (transposable element [TE] overlap). Feature‐specific callable fractions were computed per line and used as expected probabilities when calculating observed/expected enrichment of induced SNV events within the callable space (Figure S2; Table S1; Supporting Information Data S1). All callable‐space summaries are computed on a per‐line basis; we do not assume a common intersection of callable loci across all lines.

### 2.4 Reference genome and coordinate standardization

All genomic coordinates were mapped to the *S. bicolor* reference assembly (SbicolorRio_468_v2.1; Phytozome v14) (Goodstein et al., 2012). To integrate multisource annotations (e.g., TE coordinates vs. SNP calls), contig identifiers were standardized to a numeric chromosome system (Chr 1–10) using an explicit contig‐to‐chromosome mapping table (e.g., CM027680.1→1 and CM027689.1→10) implemented in the analysis scripts.

### 2.5 Computational analysis framework

All downstream statistical analyses and visualizations were implemented in Python (v3.11) using the pandas (McKinney, 2010), NumPy (Harris et al., 2020), and SciPy (Virtanen et al., 2020) libraries.

#### 2.5.1 Dose‐dependent mutation modeling

For gamma‐treated lines, we fitted an ordinary least‐squares regression of per‐line induced SNV event burden as a function of absorbed dose. Proton‐treated lines were plotted at their reported dose level (300 Gy), but no proton dose‐response model was fitted because only one proton dose was available. Regression slope, intercept, 95% confidence intervals, *p*‐value, and *R* ^2^ were reported to describe the sample‐level dose–burden relationship.

#### 2.5.2 Mutational signature analysis

To compare chemical mutagenesis mechanisms, we constructed 96‐channel trinucleotide mutational spectra for each line (Alexandrov et al., 2013). Principal component analysis (PCA) was applied to standardized spectral frequencies to assess the divergence between gamma and proton signatures.

#### 2.5.3 Macroscale genomic landscape and spatial inequality

The spatial inequality of mutation distribution was quantified by binning SNV events into 1‐Mb genomic windows. The Gini coefficient was calculated for each radiation type to measure deviation from a uniform distribution (Gini, 1912). To evaluate whether spatial inequality was driven by uneven GBS site density, we additionally counted callable GBS loci per genomic window and calculated mutation density per callable locus across 250‐kb, 500‐kb, and 1‐Mb windows. We also generated callable‐density‐weighted null distributions by sampling mutation positions according to the observed distribution of callable loci across genomic windows.

#### 2.5.4 Microscale clustering and physical track structure

To quantify spatial clustering patterns of induced mutations, we applied Ripley's *K*‐function (1D point pattern analysis) to the induced SNV coordinates (Ripley, 1976). For presentation, the *K*‐function results are shown as *H*(*r*), a transformed summary used to visualize departures from the null expectation across genomic distances. This analysis highlighted clustering scales (e.g., ∼500 kb) in the examined data. Fine‐scale clustering was visualized using Rainfall plots, plotting inter‐mutation distances to detect kataegis‐like localized hypermutation events (Nik‐Zainal et al., 2012). MBSs were operationally defined as adjacent induced SNV events separated by exactly 1 bp within the same line and chromosome; indels were not evaluated because the source dataset consisted of filtered GBS SNP loci rather than genome‐wide de novo variant calls. To validate clustering against methodological bias, we compared the cumulative distribution of inter‐mutation distances in proton and gamma lines against a null model generated by random sampling from the universe of all GBS‐accessible sites. To quantify clustered damage, high‐density mutation tracks were defined as 500‐kb genomic windows exceeding the 95th percentile of mutation density observed in the gamma‐irradiated population. To rule out simple dose‐dependence, we assessed the Spearman correlation between absorbed gamma dose and the fraction of mutations within these tracks.

#### 2.5.5 Sensitivity analysis for recurrent‐locus artifacts

To test whether short‐range clustering (±10 bp) could be inflated by recurrent, highly callable loci, genomic positions were grouped by coordinate (chromosome: position), and the number of unique lines containing each position was computed. We then recomputed per‐line cluster fractions after progressively removing loci recurring in ≥2, ≥3, ≥5, and ≥10 lines (Figure S3; Table S2; Supporting Information Data S1).

#### 2.5.6 Functional feature enrichment within callable space

Observed counts of induced SNV events falling into each genomic feature class (CDS, promoter, gene body non‐CDS, intergenic, and TE overlap) were computed from the induced event list. Expected counts were computed using feature‐specific callable fractions (denominators) estimated per line from the published SNP matrix callability (rather than genome‐length fractions). Enrichment was defined as the observed fraction divided by the expected callable fraction, and two‐sided binomial tests were used to assess deviation from callable expectations (Table S1; Supporting Information Data S1).

#### 2.5.7 Evolutionary dynamics

To test hypotheses of purifying selection versus mutation pressure, we analyzed the correlation between total mutational load and the fraction of mutations within CDS. Transition/transversion (Ts/Tv) ratios were also computed to evaluate mutation quality.

#### 2.5.8 TE analysis

TE intervals were extracted from repeat‐masked GFF annotations (Chen, 2004). The enrichment of induced mutations within TE regions was statistically tested to evaluate whether detectable induced SNV events are enriched or depleted in TE‐annotated intervals within the callable space.

### 2.6 Statistical analysis

Statistical analyses were conducted in Python (v3.11). Unless stated otherwise, tests were two‐sided with *p* < 0.05. Feature enrichment used two‐sided binomial tests; inter‐mutation distance distributions used two‐sample Kolmogorov–Smirnov tests. Correlations were evaluated using Pearson's *r* or Spearman's *ρ* as indicated. The effective sample size and unit of analysis for each statistical test are reported in the relevant results, tables, figure legends, or Supporting Information Data S1.

## 3 RESULTS

### 3.1 Mutation burden across dose levels and conserved chemical signatures

**FIGURE 1:**
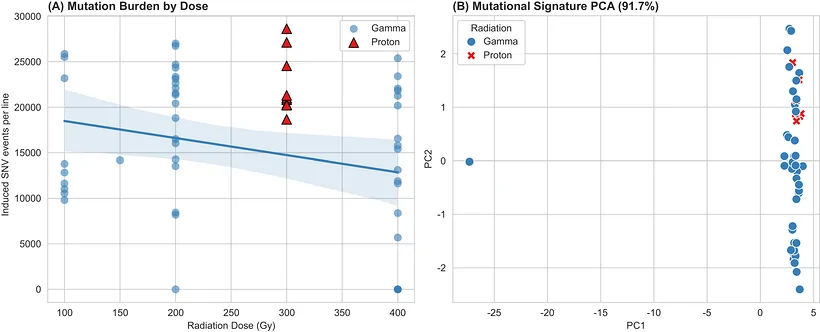
Induced single‐nucleotide variant (SNV) event burden and mutational signatures. (A) Per‐line induced SNV event burden across gamma‐ray dose levels and the single proton‐beam dose level. Gamma‐treated lines show substantial within‐dose variation; the fitted sample‐level regression is reported descriptively in the text and Supporting Information Data S1, and the slope was not significant at α = 0.05. Proton‐treated lines are shown at 300 Gy, but proton dose‐response parameters were not estimated because only one proton dose was available. (B) Principal component analysis (PCA) of 96‐channel mutational signatures. Gamma‐ and proton‐treated lines occupy overlapping chemical space, indicating broadly similar base‐substitution spectra despite differences in spatial distribution.

To establish a quantitative baseline for the dataset, we first summarized induced mutation burden across radiation treatments (Figure 1A). Using our pairwise mutant–parent calling pipeline, we identified a total of 946,537 putative induced SNV events, counted cumulatively across all line–locus comparisons, across the 59 mutagenized lines. This burden should not be interpreted as a genome‐wide de novo mutation rate. It represents the cumulative number of mutant–parent genotype differences detected at loci already present in the filtered GBS SNP panel, so the magnitude can be inflated relative to whole‐genome sequencing (WGS)‐based de novo mutation estimates by reduced‐representation ascertainment, residual segregating variation, heterozygous/ambiguous genotype calls, and genotype‐call uncertainty. In the regenerated sample‐level analysis, induced SNV burden varied substantially among gamma‐treated lines across the 100–400 Gy dose range. The fitted gamma regression had an intercept of 20,354.5 events and a slope of −18.7 events Gy^−1^, with limited explanatory power (*R* ^2^ = 0.076; *p* = 0.057; 95% confidence intervals reported in Supporting Information Data S1). We therefore treat the dose–burden regression as descriptive rather than as evidence for a strong linear dose‐response. Proton‐treated lines were observed only at a single dose level (300 Gy), and therefore proton‐specific dose‐response parameters could not be estimated. At 300 Gy, proton‐treated lines fell within the upper range of the gamma‐treated mutation burdens (Figure 1A), but additional matched proton and gamma dose levels would be required to evaluate relative mutagenic efficiency per unit dose.

Despite this difference in intensity, the underlying chemical mechanisms of mutagenesis appeared remarkably conserved. PCA of 96‐channel trinucleotide mutational spectra revealed largely overlapping clusters for gamma and proton lines, with only minor separation along the first principal component (PC1) (Figure 1B). Both radiation types were dominated by C > T and T > C transitions in similar sequence contexts, suggesting that the fundamental biochemical lesions are shared regardless of the radiation source. Thus, the qualitative distinction between proton and gamma mutagenesis is not driven by what kind of mutations are made, but likely by where they are made.

### 3.2 Proton irradiation imprints a spatially unequal, spike‐like genomic landscape

**FIGURE 2:**
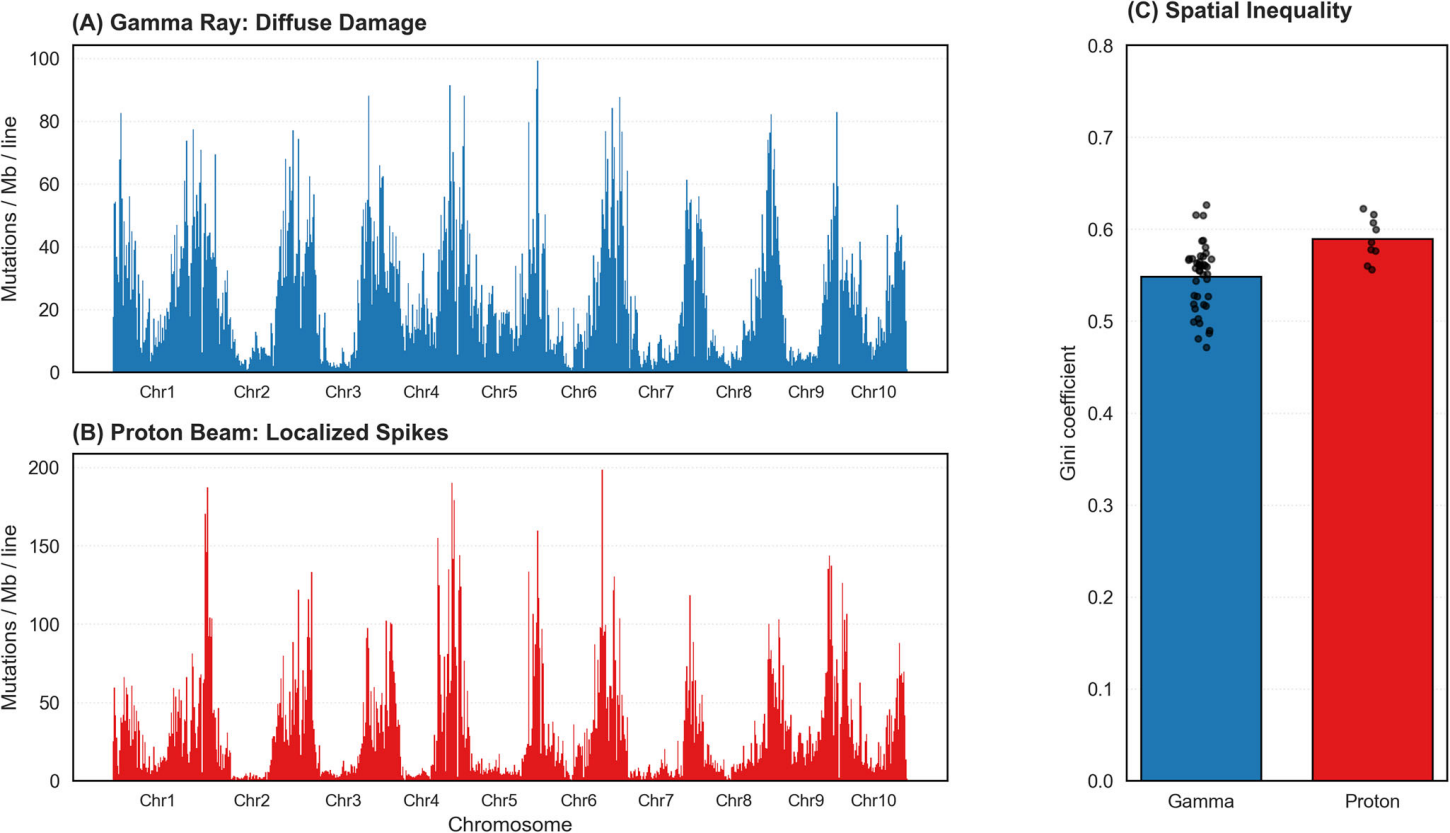
Macroscale genomic geometry of damage. (A) Chromosomal mutation landscape for gamma‐irradiated lines, showing a more diffuse distribution of mutation density (mutations/Mb). (B) Chromosomal mutation landscape for proton‐irradiated lines, revealing distinct, high‐density mutational “spikes” indicative of localized damage concentration. (C) Gini coefficient analysis quantifying spatial inequality. Proton irradiation results in higher Gini values, reflecting a more clustered and unequal distribution of mutations compared to gamma irradiation.

Having established that mutational quantity and quality are broadly comparable, we next investigated the spatial distribution of these mutations at the chromosomal scale. We binned induced SNV events into 1 Mb genomic windows to construct a macroscale mutation landscape. Gamma‐irradiated lines displayed a relatively diffuse distribution across chromosomes within the callable locus set (Figure 2A). In sharp contrast, proton‐irradiated lines exhibited a highly unequal landscape characterized by dramatic “spikes,” where specific 1 Mb windows contained mutation densities several fold higher than the genomic background (Figure 2B). These spikes were consistently observed across multiple proton lines, indicative of localized high‐energy deposition events.

### 3.3 Spatial inequality and localized hotspots

To quantify the observed spatial inequality, we calculated statistical metrics for mutation density distributions (Table 1). Proton landscapes yielded a higher Gini coefficient (∼0.53) compared to gamma landscapes (∼0.44), consistent with a more concentrated and less uniform distribution of detectable mutation events (Figure 2C). This “spikiness” was further evidenced by the intensity of mutational clusters; the maximum mutation density in proton lines reached 220.3 mutations/Mb, about 1.7‐fold higher than the peak density observed in gamma lines (131.6 mutations/Mb). Moreover, we identified 15 distinct mutational hotspots in proton‐irradiated genomes, whereas only six hotspots were found in gamma‐irradiated genomes despite the larger sample size of the gamma group.

**TABLE 1:** Spatial statistics summary: Summary of Gini coefficients, maximum bin densities, and hotspot counts comparing gamma and proton treatments.

Metric	Gamma ray	Proton beam	Note
Gini coefficient	0.44	0.53	Higher inequality in proton
Max mutation density (muts/Mb)	131.6	220.3	Higher peak intensity in proton
Hotspot count (Mean + 3 SD)	6	15	2.5‐fold more hotspots in proton
Coefficient of variation (CV)	0.8	1.08	Greater dispersion in proton
Mean mutation density (muts/Mb)	24.4	31	Baseline mutation rate

*Note*: Mean mutation density (mutations/Mb) was computed as the mean of 1‐Mb bin counts divided by the number of lines in each radiation group (i.e., mutations per 1‐Mb bin per sample), across Chr1–10. Hotspots were defined as 1‐Mb windows with mutation density greater than the group mean plus three standard deviations.

These metrics provide quantitative support, in this dataset, for more unequal window‐level mutation distributions in proton‐treated lines compared to gamma‐treated lines. To evaluate whether these window‐level patterns were driven by uneven GBS site density, we additionally normalized mutation counts by callable‐site counts within genomic windows. Proton‐treated lines retained higher mutation density per callable locus across 250‐kb, 500‐kb, and 1‐Mb windows (Supporting Information Data S1). We also generated callable‐density‐weighted spatial null distributions, in which mutation positions were sampled according to the distribution of callable loci. Observed Gini coefficients exceeded the callable‐density‐weighted null expectation in both radiation groups, with a larger observed‐minus‐null difference in proton‐treated lines. However, differences in normalized Gini metrics were reduced relative to the unnormalized analysis, indicating that callable‐site density contributes to, but does not fully explain, the observed spatial inequality.

### 3.4 Microscale biophysics reveals track‐structure clustering and complex damage

**FIGURE 3:**
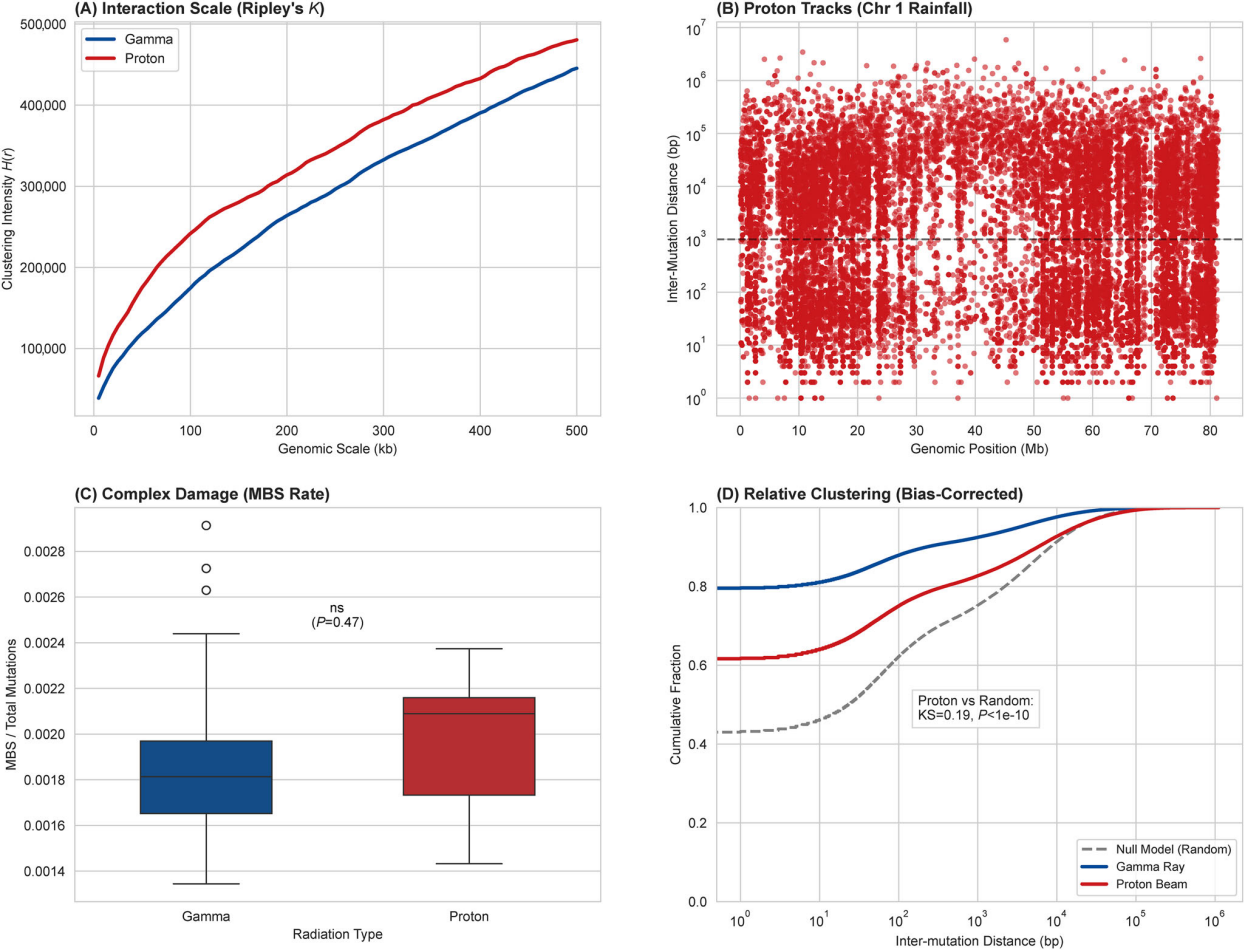
Microscale biophysics and track structure. (A) Ripley's *K* function analysis (*H*(*r*)) showing elevated clustering in proton‐treated lines across evaluated sub‐megabase scales; 500 kb was used as an operational track window. (B) Rainfall plot of inter‐mutation distances on Chr 1 for a representative proton line showing dense tracks. (C) Comparison of multi‐base substitution (MBS) rates; no significant difference was detected between radiation groups (*p* = 0.47). (D) Cumulative distribution of inter‐mutation distances. Proton‐induced mutations (red) exhibit significantly shorter distances compared to the random null model derived from all genotyping‐by‐sequencing (GBS)‐accessible sites (dashed gray), supporting that clustering patterns are not fully explained by random sampling from callable loci (*p* $<10^{-10}$, Kolmogorov–Smirnov [KS] test), although locus‐level artifacts cannot be entirely excluded in reduced‐representation data.

To resolve the fine structure of these genomic spikes, we zoomed in to the microscale using point‐pattern analysis. We applied a one‐dimensional Ripley's *K* function to estimate the characteristic clustering scale. While both radiation types showed some clustering, proton lines exhibited higher clustering intensity (*H*(*r*)) across the evaluated sub‐megabase scales up to 500 kb (Figure 3A). Because clustering remained elevated at this upper evaluated scale, we used 500‐kb windows as an operational track definition, rather than as evidence for a discrete physical boundary.

Direct visualization of inter‐mutation distances via rainfall plots further corroborated this track structure. For example, on Chromosome 1, proton‐treated lines displayed distinct “kataegis‐like” vertical bands where successive mutations were separated by less than 1 kb (Figure 3B); this term is used only as a visual analogy for localized clustering and does not imply a mutational process mediated by apolipoprotein B mRNA editing enzyme, catalytic polypeptide‐like (APOBEC) family cytidine deaminases. These dense clusters are consistent with localized clustering expected from charged‐particle track structure. As a descriptive proxy for complex local sequence changes, we analyzed the rate of MBS. MBS rates did not differ significantly between gamma‐ and proton‐treated lines (Figure 3C; Supporting Information Data S1), indicating that this metric does not provide strong evidence for a radiation‐type difference in complex base‐substitution events in the present dataset. Crucially, when we normalized inter‐mutation distances against the background of all GBS‐detectable loci, proton‐induced mutations exhibited a significant deviation from the random null model toward shorter distances (*p* $<10^{-10}$), supporting that clustering patterns are not fully explained by random sampling from callable loci; however, reduced‐representation data cannot fully exclude alignment and locus‐level artifacts, motivating the recurrent‐locus sensitivity analyses below (Figure 3D). Quantitatively, proton‐irradiated genomes concentrated ∼37% of their total mutational burden into high‐density tracks (500‐kb scale), compared to only ∼21% in gamma‐irradiated genomes. This nearly two‐fold difference is consistent with a greater concentration of detectable proton‐associated mutation events within high‐density windows, rather than a uniformly diffuse accumulation pattern.

### 3.5 Validation of track structure independence from dose

A potential confounding factor is whether the observed clustering in proton‐treated lines reflects radiation type or simply higher mutation burden. We first analyzed the relationship between absorbed gamma‐ray dose and the fraction of mutations falling into high‐density 500‐kb tracks across gamma‐treated lines and found no significant correlation (Spearman's *ρ* = −0.10, *p* = 0.52; Figure S4). We further compared proton‐treated lines with the highest‐load tertile of gamma‐treated lines. Proton‐treated lines had a similar or slightly lower mean total event burden than the high‐load gamma tertile, but retained a significantly higher short‐range clustering fraction (Supporting Information Data S1). By contrast, the 500‐kb track fraction was only modestly higher in proton‐treated lines than in the high‐load gamma tertile and was not statistically different. These results support proton‐associated clustering most strongly at the short‐range scale, while the 500‐kb track definition should be interpreted as an operational summary of high‐density windows rather than as a definitive physical boundary. We also evaluated alternative window sizes and track thresholds as sensitivity analyses; these results are reported in Supporting Information Data S1 and were used to interpret the 500‐kb definition as an operational summary rather than a discrete biological boundary.

### 3.6 Clustering is robust to recurrent‐locus filtering

To assess whether short‐range clustering could be driven by a limited set of recurrent loci (e.g., highly callable sites), we recalculated per‐line cluster fractions after removing loci recurring across multiple lines (≥2, ≥3, ≥5, and ≥10 lines). Recurrent‐locus filtering reduced the mean proton cluster fraction more strongly than the mean gamma cluster fraction, indicating that recurrent high‐callability loci contribute to the raw short‐range signal (Figure S3; Table S2). However, cluster fractions were not eliminated across filtering thresholds, and under the most stringent filter (≥10 lines) proton‐treated lines again showed a higher mean cluster fraction than gamma‐treated lines. These results suggest that recurrent loci contribute to, but do not fully explain, short‐range clustering in the reduced‐representation dataset.

### 3.7 Callable‐space‐corrected feature enrichment

**FIGURE 4:**
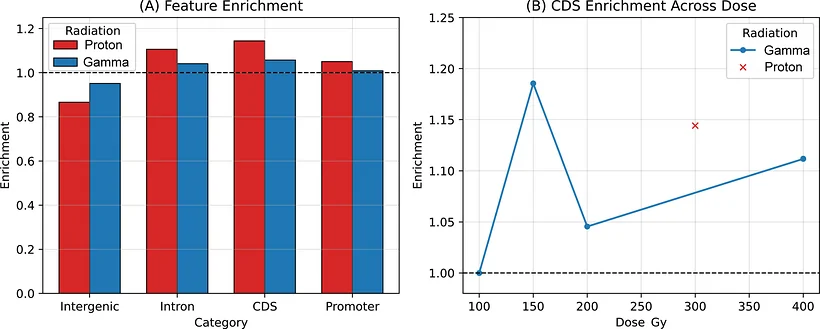
Callable‐space‐corrected feature enrichment. (A) Enrichment of induced single‐nucleotide variant (SNV) events across genomic features. Values above 1.0 indicate enrichment relative to the callable‐space expectation, and values below 1.0 indicate depletion. (B) Coding sequence (CDS) enrichment across gamma‐ray dose levels and the single proton‐beam dose level.

We next quantified feature enrichment of induced SNV events using the callable‐space framework to account for nonuniform GBS sampling. Expected feature proportions were derived from feature‐specific callable denominators estimated per line from the published SNP matrix, and enrichment was computed as observed/expected within this GBS‐accessible space (Table S1; Supporting Information Data S1). Using callable‐corrected expectations, CDS and promoter regions showed enrichment values above 1.0, whereas intergenic regions and TE‐overlap space were depleted for both radiation types, with a stronger gene‐proximal skew in proton‐treated lines (Figure 4A; Table 2; Figure S2). These results indicate that coding‐ and promoter‐proximal categories were not depleted within the callable locus set, but they do not by themselves demonstrate active shielding or tolerance mechanisms. To contextualize this pattern, we also correlated mutation density with gene density across 1‐Mb windows. Gamma‐induced mutations showed a stronger positive correlation with gene density (Pearson *r* = 0.78), whereas proton‐induced mutations showed a weaker correlation (*r* = 0.69), consistent with localized high‐density windows partially perturbing the gene‐density‐correlated distribution (Figure S5).

**TABLE 2:** Callable‐space‐corrected functional enrichment statistics.

Radiation	Feature	Observed	Expected (callable‐corrected)	Enrichment	*p*‐value
Proton	CDS	45,443	39,719.32	1.144103	<1E‐100
Proton	Promoter	23,848	22,708.15	1.050196	1.54E‐15
Proton	Intron (gene body, non‐CDS)	54,906	49,656.84	1.105709	<1E‐100
Proton	Intergenic	78,052	90,164.7	0.86566	<1E‐100
Proton	TE overlap^a^	42,216	55,023	0.767243	<1E‐100
Gamma	CDS	152,837	144,624.5	1.056785	<1E‐100
Gamma	Promoter	83,942	83,246.07	1.00836	1.06E‐02
Gamma	Intron (gene body, non‐CDS)	188,809	181,411	1.040781	4.68E‐88
Gamma	Intergenic	316,574	332,880.4	0.951014	<1E‐100
Gamma	TE overlap^a^	184,535	204,831.5	0.900911	<1E‐100

*Note*: Observed and expected induced single‐nucleotide variant (SNV) event counts were calculated for each genomic feature using feature‐specific callable denominators. An enrichment ratio (Obs/Exp) > 1.0 indicates enrichment within the callable space, while a ratio <1.0 indicates depletion. *p*‐values were calculated using two‐sided binomial tests. Counts are restricted to annotated loci on Chr1–10 within the GBS‐callable dataset and therefore do not necessarily sum to the total induced‐event count reported in the text.

Abbreviations: CDS, coding sequence; TE, transposable element.

^a^ TE overlap is not mutually exclusive with the CDS; promoter; gene body, non‐CDS; or intergenic categories.

We further examined whether CDS enrichment changed across the gamma dose gradient. CDS enrichment did not show an obvious decrease with increasing dose and remained above the callable‐space expectation across the dose levels examined (Figure 4B; Table 2). Given the reduced‐representation design and the single proton dose, we interpret this pattern as a lack of detectable CDS depletion within the callable locus set, rather than as direct evidence for active shielding or tolerance mechanisms.

### 3.8 Relationship between mutational load and coding‐region fraction

**FIGURE 5:**
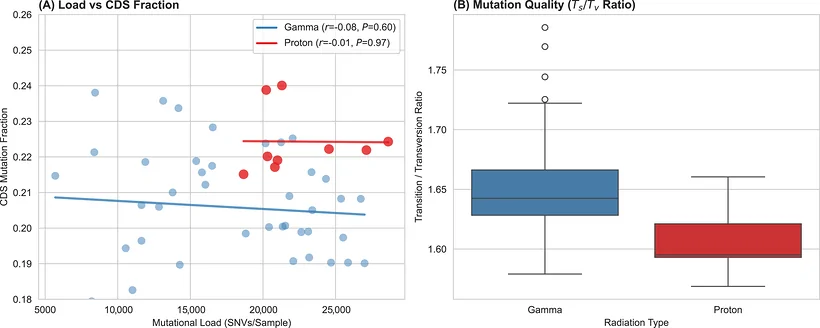
Relationship between mutational load and mutational quality. (A) Correlation between total mutational load and coding sequence (CDS) mutation fraction. (B) Comparison of transition/transversion (Ts/Tv) ratios. Proton lines exhibited a small shift toward lower Ts/Tv values compared with gamma lines, while overall ratios remained broadly comparable between radiation types.

Finally, we examined whether higher mutational load was associated with a reduced fraction of coding‐region mutations. If strong purifying selection were evident within this dataset, lines with higher total mutational loads would be expected to show a lower fraction of mutations in coding regions. We did not detect a significant negative relationship between total mutational load and CDS mutation fraction in either gamma‐ or proton‐treated lines (Figure 5A; Table 3). Given the limited sample size for proton‐treated lines and the relatively narrow proton load range, this result is consistent with a weak or undetectable purifying selection signal in the present dataset, rather than proof of evolutionary indifference.

**TABLE 3:** Load–coding sequence (CDS) association and transposable element (TE) enrichment statistics. Summary of per‐line mutational loads, CDS fractions, transition/transversion (Ts/Tv) ratios, and TE enrichment data.

Metric	Gamma ray	Proton beam	Interpretation
Correlation: Load vs. CDS%	*r *= −0.08 (*p* = 0.60)	*r* = −0.01 (*p* = 0.97)	No negative load–CDS association detected
TE overlap enrichment (callable‐corrected)	0.90	0.77	Depletion in TE‐overlap intervals within callable space
Mutation quality (Ts/Tv ratio)	1.65	1.61	Slight skew to transversions in proton

Additionally, we compared the Ts/Tv ratio as a metric of mutation quality. The Ts/Tv ratios were broadly similar between gamma and proton lines (Figure 5B; Table 3), though proton lines exhibited a slight skew toward lower values (1.61 vs. 1.65 for gamma). Given the small magnitude of this difference, we interpret Ts/Tv ratios as broadly comparable between radiation types. However, the overall stability of this ratio suggests that while the spatial delivery of damage differs, the qualitative nature of genetic variation remains largely comparable. Complementary analysis of TE‐overlap intervals showed depletion relative to the callable‐space expectation, particularly for proton‐treated lines (Table 3), consistent with a gene‐proximal skew among detectable events within the callable locus set.

## 4 DISCUSSION

### 4.1 Proton irradiation imprints a distinct biophysical geometry of damage

The primary finding of this study is that while proton‐beam and gamma‐ray mutagenesis in *S. bicolor* appear quantitatively similar in terms of total mutational burden and chemical signatures (Figure 1), they differ in their spatial geometry of damage. Gamma irradiation, a low‐LET source, deposits energy diffusely, resulting in a more diffuse mutation distribution within the callable locus set (Figure 2A). In contrast, proton irradiation, a charged‐particle source with track‐structured energy deposition, creates a highly unequal, “spiky” distribution of mutations (Figure 2B), summarized by higher Gini coefficients within the callable locus set (Figure 2C). This observation aligns with biophysical models of charged particle tracks, which predict that high‐LET radiation deposits energy in dense ionization clusters rather than random scatter (Goodhead, 1994; Nikjoo et al., 2016). Recent WGS studies in Arabidopsis and rice have consistently supported this LET‐dependent mutational dichotomy, demonstrating that heavy‐ion beams (e.g., carbon, argon) induce complex structural variations and large deletions (>100 bp) at frequencies significantly higher than low‐LET radiation (Hirano et al., 2012; Ichida et al., 2019; Kazama et al., 2017; Ren et al., 2023).

Our microscale analysis further resolves this geometry. The characteristic clustering scale of ∼500 kb identified by Ripley's *K* analysis (Figure 3A) is further substantiated by our track‐calling analysis, which revealed that a substantial fraction (∼37%) of proton‐induced mutations are sequestered within these high‐density domains. This suggests that proton tracks do not merely create isolated lesions but may traverse chromosomal subregions on the scale of hundreds of kilobases. The “rainfall” pattern of localized hypermutation (Figure 3B) resembles clustered mutational phenomena observed in other systems (Nik‐Zainal et al., 2012), consistent with such track‐structure‐driven clustering in plant radiation mutagenesis. Although MBS rates were not statistically different between radiation groups (Figure 3C), this metric provides a descriptive proxy for local sequence complexity, a feature often considered in discussions of clustered DNA lesion repair (Sage & Harrison, 2011). Conversely, recent WGS comparisons in Arabidopsis and cowpea confirm that gamma‐rays predominantly induce single‐base substitutions and small indels with limited clustering, reinforcing the unique structural footprint of particle radiation (Du et al., 2022; Jo & Kim, 2019; Punniyamoorthy & Souframanien, 2024). Thus, we propose that a distinguishing feature of proton mutagenesis in this dataset is not its chemical specificity, but its spatial architecture, a “geometry of damage” that concentrates mutational load into specific genomic loci.

### 4.2 Validation via gene density correlation

To further contextualize the spatial distribution of detectable SNV events, we correlated mutational density with gene density derived from the genome annotation (Figure S5). Gamma‐induced mutations showed a strong correlation with gene density (Pearson *r* = 0.78), consistent with a gene‐density‐correlated distribution within the callable locus set. In contrast, proton‐induced mutations exhibited a weaker correlation (*r* = 0.69), suggesting that their distribution is not solely governed by gene density but is distorted by localized clustering events (spikes). This deviation is consistent with proton‐associated physical track structure producing patterns that depart from gene‐density‐correlated expectations within the callable space, concentrating the burden in specific focal domains beyond what is expected from gene density alone. This pattern aligns with recent observations in mammalian systems that high‐LET radiation interacts preferentially with biologically active, euchromatic sub‐compartments (Friedman et al., 2016; Venkatesh et al., 2016).

### 4.3 Feature enrichment within the GBS‐callable space

The functional enrichment analysis should be interpreted within the constraints of the GBS‐callable locus set. CDS and promoter‐proximal categories were enriched relative to callable‐space expectations, whereas intergenic and TE‐overlap intervals were depleted (Figure 4; Table 2). This pattern indicates that detectable induced SNV events were not depleted from coding‐ or promoter‐proximal regions within the reduced‐representation dataset. However, because GBS preferentially samples gene‐rich, hypomethylated regions (Elshire et al., 2011), these results should not be interpreted as direct evidence for genome‐wide targeting, active shielding, or tolerance mechanisms. Instead, they support a more limited conclusion: within the GBS‐callable space, functional categories remained represented among detectable induced mutation events. Plant genome architecture and DNA‐repair biology may influence survival after irradiation (Hefner et al., 2003; Miller et al., 2021; Paterson et al., 2009; Spampinato, 2017; van de Kamp et al., 2021; Zhao et al., 2020). However, the present GBS data do not directly test these mechanisms.

### 4.4 Load‐coding‐region relationship in surviving mutant lines

Classical evolutionary theory predicts that purifying selection can reduce the frequency of deleterious coding‐region mutations over time (Kimura, 1985; Nei, 2005). In this surviving mutant panel, we did not detect a significant negative relationship between total mutational load and the fraction of coding‐region mutations in either radiation group (Figure 5A; Table 3). This result suggests that, within the current sample size and callable locus set, a purifying‐selection signal against coding‐region mutation burden was weak or not detectable. However, the proton group contained only nine lines and covered a relatively narrow burden range, limiting statistical power to detect moderate correlations. Therefore, this analysis should be viewed as a descriptive assessment of surviving mutant lines rather than as evidence that selection was absent or overwhelmed. The broadly similar Ts/Tv ratios further support the separate conclusion that substitution spectra were broadly comparable between radiation types despite differences in spatial distribution (Figure 5B).

### 4.5 Revisiting McClintock: Spatial clustering and stress‐induced genome instability

The spatially concentrated mutation patterns observed in proton‐treated lines can be placed in the broader context of stress‐induced genome instability, as framed by McClintock's concept of “genomic shock” (Fedoroff, 2012; McClintock, 1984). In this study, however, “genomic shock” should be interpreted as a conceptual framework rather than as direct evidence for genome‐wide restructuring. The present GBS dataset does not directly measure structural rearrangements, transcriptional responses, chromatin remodeling, or transposition events. Instead, our results show that detectable SNV events in proton‐treated lines are concentrated into high‐density spatial domains within the callable locus set, suggesting that proton‐beam irradiation is associated with a distinct spatial pattern of genomic perturbation.

Our TE‐overlap analysis showed depletion of detectable SNV events in TE‐annotated intervals, particularly in proton‐treated lines (Table 3), but this result is likely influenced by the reduced representation of methylated and repeat‐rich regions in GBS data. Therefore, the depletion of SNV events in TE‐overlap space should not be interpreted as evidence against TE‐related stress responses. In a previous analysis of this same population, TE‐associated functional terms, including DNA integration and RNA‐directed DNA polymerase, were enriched in predictive models of agronomic and phenolic traits (Ahn et al., 2025). In addition, previous studies have reported that physical stresses, including ion‐beam or laser irradiation, can alter gene expression and activate TEs such as *mPing* and LTR retrotransposons in rice (Li et al., 2017; Ya et al., 2012).

Together, these observations support a restrained model in which proton‐associated high‐density mutation tracks may represent one measurable component of a broader stress‐induced genome‐instability response. This model remains hypothesis‐generating: direct testing will require WGS, structural variant analysis, and transcriptomic or epigenomic profiling across matched proton and gamma dose series. Within this limitation, the observed spatial concentration of detectable mutation events is consistent with the potential utility of proton‐beam irradiation for generating localized, high‐density mutational variation in mutation‐breeding contexts (Oladosu et al., 2016).

### 4.6 Robustness of spatial patterns against methodological bias

A potential caveat of our study is the reliance on GBS, which preferentially samples gene‐rich, hypomethylated regions (Elshire et al., 2011). We therefore interpret all spatial analyses within the GBS‐callable locus set and do not claim genome‐wide mutation‐rate estimates. The additional callable‐density normalization and callable‐weighted null analyses reduce, but cannot eliminate, the possibility that uneven callable‐site density contributes to apparent clustering. Within this constrained locus set, gamma‐induced mutations were closer to the callable‐loci null expectation, whereas proton‐induced mutations showed stronger deviation toward short inter‐mutation distances (Figure 3D). Furthermore, proton mutation density showed a lower correlation with gene density (*r* = 0.69) compared to gamma rays (*r* = 0.78), suggesting distortion by localized clustering events (Figure S5). Finally, we confirmed that the high frequency of track‐like clusters in proton lines is not an artifact of dose, as gamma lines showed no correlation between dose and track fraction within the tested range (Figure S4). Collectively, these validations support the interpretation that the track‐like clustering of proton damage is not fully explained by the assessed biases and persists under multiple sensitivity checks.

### 4.7 Conclusion: A unified empirical model of proton‐beam mutagenesis

**FIGURE 6:**
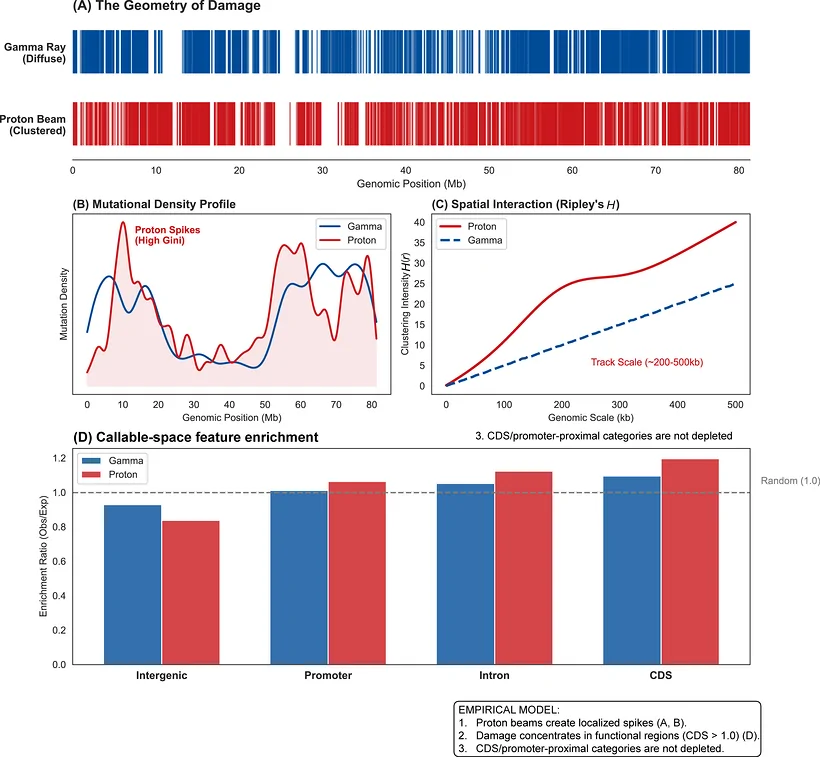
Empirical model of spatially structured mutation patterns associated with proton‐beam irradiation. This conceptual summary is a quantitative synthesis derived directly from the observed data distributions on a representative chromosome (Chr 1) and genome‐wide statistics. (A) The physical trigger (geometry of damage): Barcode plot of actual mutation positions (Chr 1) for representative gamma (blue) and proton (red) lines. Proton irradiation leaves distinct “track‐like” footprints, characterized by localized bursts of mutations separated by long gaps. These tracks summarize the spatially clustered mutation pattern observed in the representative proton‐treated line. (B) Mutational density profile: Kernel density estimation (KDE) reveals the high‐amplitude fluctuations (“spikiness”) of proton mutation density, consistent with the high Gini coefficient observed in Figure 2. (C) Spatial interaction scale: Schematic representation of Ripley's *H* function trends. Proton‐treated lines show elevated sub‐megabase clustering, summarized here at the ∼200–500 kb scale. (D) Callable‐space feature enrichment: Observed/expected enrichment ratios for genomic features. Values above 1.0 indicate enrichment relative to callable‐space expectations, whereas values below 1.0 indicate depletion. This panel summarizes detectable feature enrichment patterns within the genotyping‐by‐sequencing (GBS)‐callable locus set and should not be interpreted as direct evidence for genome‐wide shielding or tolerance mechanisms.

By integrating multi‐scale spatial statistics with functional genomic analysis, we propose a unified empirical model for proton‐beam mutagenesis (Figure 6). Unlike the stochastic and diffuse deposition of low‐LET gamma rays, proton beams act as structured physical agents that imprint a distinct “geometry of damage” on the genome. This geometry is defined by (1) localized, high‐density mutational tracks (Figure 6A,B); (2) a characteristic physical interaction scale of hundreds of kilobases (Figure 6C); and (3) enrichment of detectable mutation events in gene‐proximal categories within the GBS‐callable space (Figure 6D). These findings support a model in which, within the GBS‐callable portion of the sorghum genome, proton‐beam irradiation is associated with a more spatially structured distribution of detectable mutation events than gamma irradiation. Rather than demonstrating genome‐wide protection or tolerance mechanisms directly, the results suggest that proton‐beam irradiation is associated with localized, high‐density mutation patterns that may be useful for expanding allelic diversity in mutation‐breeding contexts. WGS across matched proton and gamma dose series will be required to test this model more directly.

## AUTHOR CONTRIBUTIONS

**Ezekiel Ahn**: Conceptualization; formal analysis; funding acquisition; investigation; methodology; supervision; writing—original draft. **Insuck Baek**: Formal analysis; resources; writing—review and editing. **Seunghyun Lim**: Formal analysis; software; validation; writing—review and editing. **Louis K. Prom**: Resources; writing—review and editing. **Moon S. Kim**: Resources; writing—review and editing. **Lyndel W. Meinhardt**: Funding acquisition; resources; supervision; writing—review and editing. **Clint Magill**: Methodology; resources; writing—review and editing.

## CONFLICT OF INTEREST STATEMENT

The authors declare no conflicts of interest.

## DECLARATION OF GENERATIVE AI AND AI‐ASSISTED TECHNOLOGIES

During the preparation of this work, the authors used ChatGPT (OpenAI) to improve the readability and language of the manuscript, and GitHub Copilot to assist with Python code refactoring and optimization. After using these tools, the authors reviewed and edited the content as needed and take full responsibility for the content of the publication.

## Supporting information



Table S1. Callable‐space–corrected enrichment of induced SNV events across genomic features.
Table S2. Sensitivity of short‐range clustering to recurrent‐locus filtering.

Dataset S1. Master Excel workbook containing full statistical outputs and per‐line results.

Figure S1. Callability QC within the GBS‐accessible space.

Figure S2. Feature‐specific callable denominators used for callable‐space correction.

Figure S3. Sensitivity of short‐range clustering to recurrent‐locus filtering.

Figure S4. Dose‐independence of mutational clustering in gamma‐irradiated lines.

Figure S5. Correlation between induced mutation density and genomic gene density.

## Data Availability

The published filtered GBS genotype matrix and phenotypic data analyzed in this study were derived from the publicly available dataset by Lee et al. (2023). The *Sorghum bicolor* reference genome (SbicolorRio_468_v2.1) and gene annotations used for mapping are available through the Phytozome portal (Goodstein et al., 2012). All derived datasets generated in this study, including induced mutation calls, spatial statistics, track analysis results, and functional enrichment tables, are provided in the Supporting Information accompanying this article (Supporting Information Data S1). The custom Python computational pipeline developed for spatial geometry analysis and track calling is openly available on GitHub at https://github.com/EJSAHN/Sorghum‐Proton‐Mutagenesis. Any additional intermediate files are available from the corresponding author upon reasonable request.
