# Supplementary material for: High‐density mutation tracks are associated with proton‐beam irradiation patterns in Sorghum bicolor
Source: Plant Genome. 2026 Jun 29;19(3):e70267. doi: 10.1002/tpg2.70267 (PMC13315512; doi:10.1002/tpg2.70267)

Fig Sx. Callability QC within the GBS-accessible space

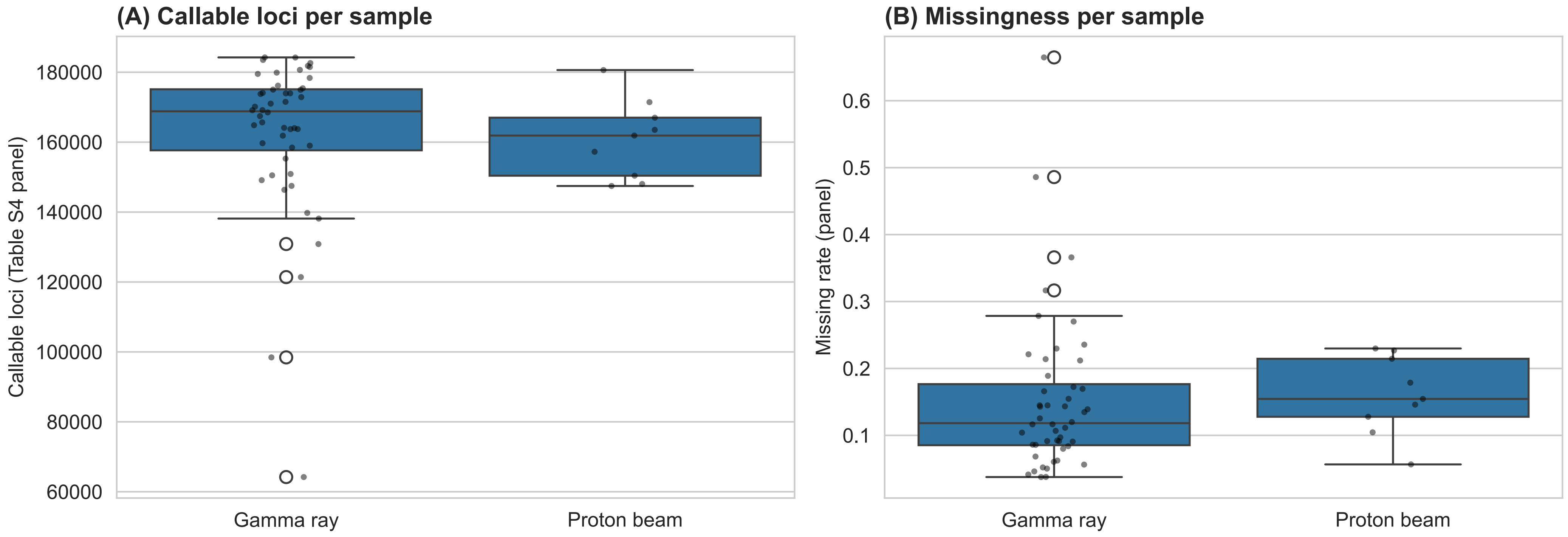

Supplement: Supplementary file 1 [file TPG2-19-e70267-s003.pdf]
