# Supplementary material for: High‐density mutation tracks are associated with proton‐beam irradiation patterns in Sorghum bicolor
Source: Plant Genome. 2026 Jun 29;19(3):e70267. doi: 10.1002/tpg2.70267 (PMC13315512; doi:10.1002/tpg2.70267)
Supplement: Supplementary file 2 [file TPG2-19-e70267-s004.pdf]

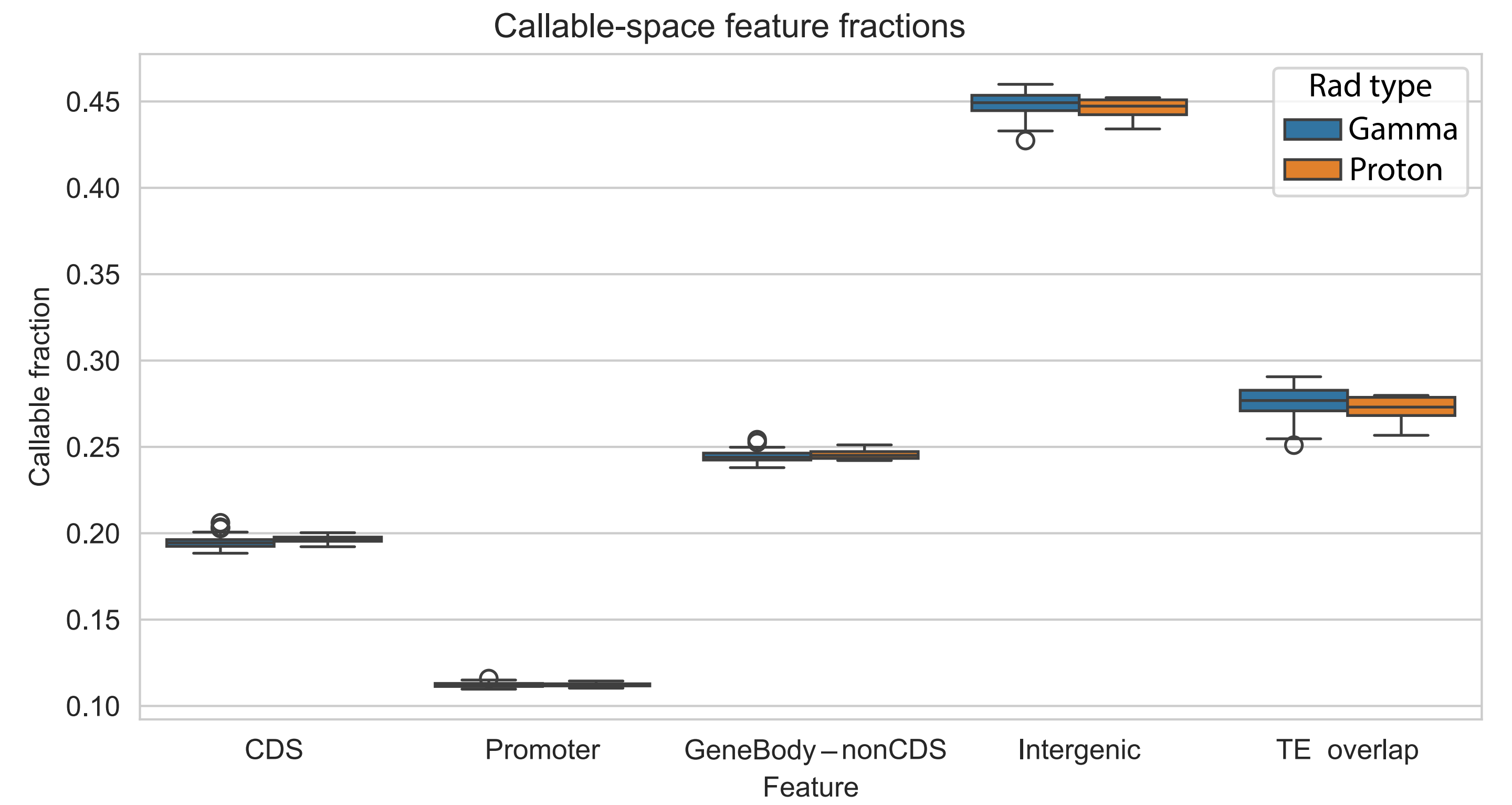

Callable-space feature fractions
Callable fraction
0.45
0.40
0.35
0.30
0.25
0.20
0.15
0.10
Rad type
Gamma
Proton
CDS
Promoter
GeneBody – nonCDS
Intergenic
TE overlap
Feature
