# Supplementary material for: High‐density mutation tracks are associated with proton‐beam irradiation patterns in Sorghum bicolor
Source: Plant Genome. 2026 Jun 29;19(3):e70267. doi: 10.1002/tpg2.70267 (PMC13315512; doi:10.1002/tpg2.70267)
Supplement: Supplementary file 3 [file TPG2-19-e70267-s005.pdf]

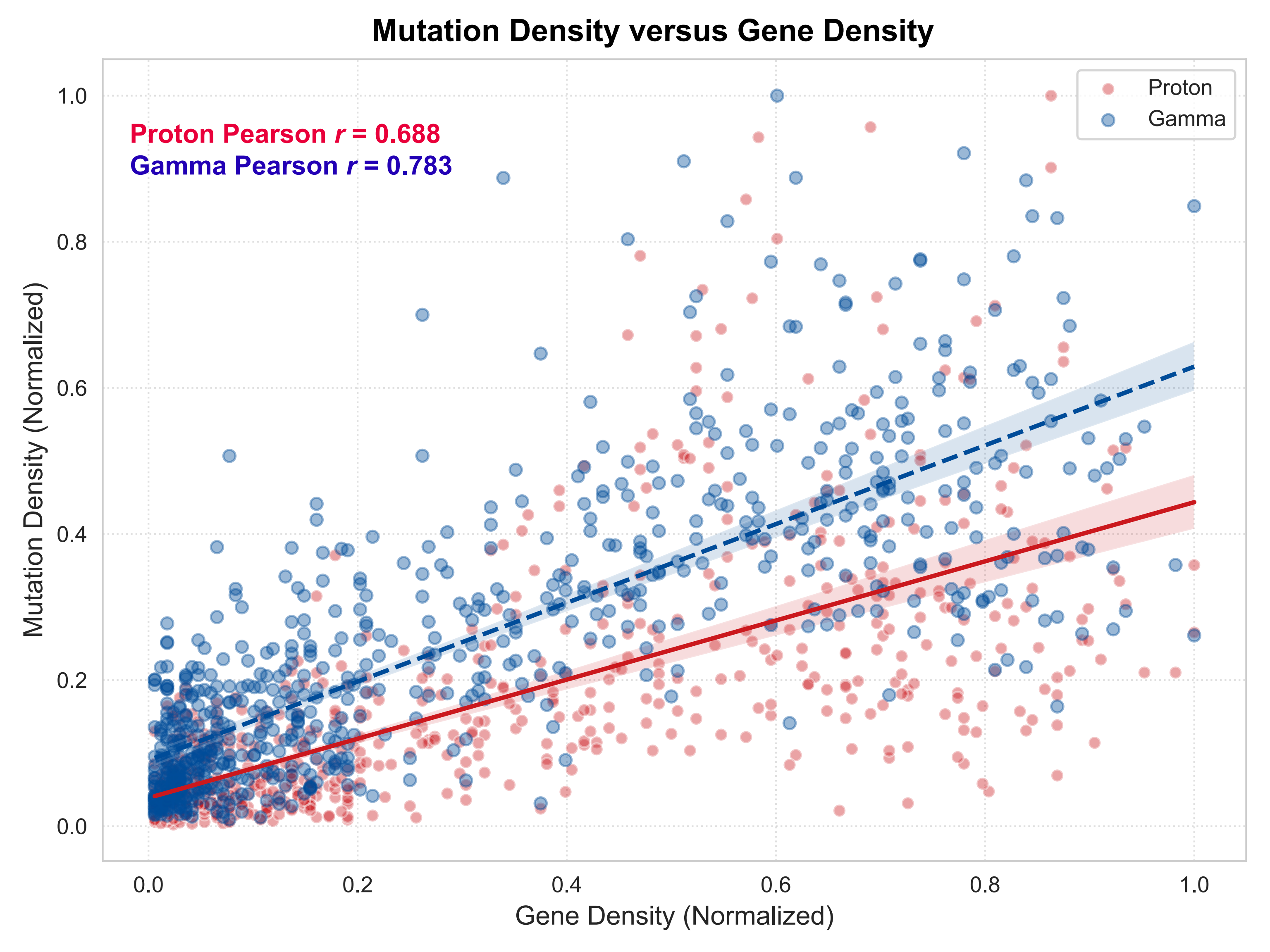

Mutation Density versus Gene Density
Proton Pearson r = 0.688
Gamma Pearson r = 0.783
Proton
Gamma
Mutation Density (Normalized)
Gene Density (Normalized)
0.0
0.2
0.4
0.6
0.8
1.0
