# Supplementary material for: High‐density mutation tracks are associated with proton‐beam irradiation patterns in Sorghum bicolor
Source: Plant Genome. 2026 Jun 29;19(3):e70267. doi: 10.1002/tpg2.70267 (PMC13315512; doi:10.1002/tpg2.70267)
Supplement: Supplementary file 4 [file TPG2-19-e70267-s006.pdf]

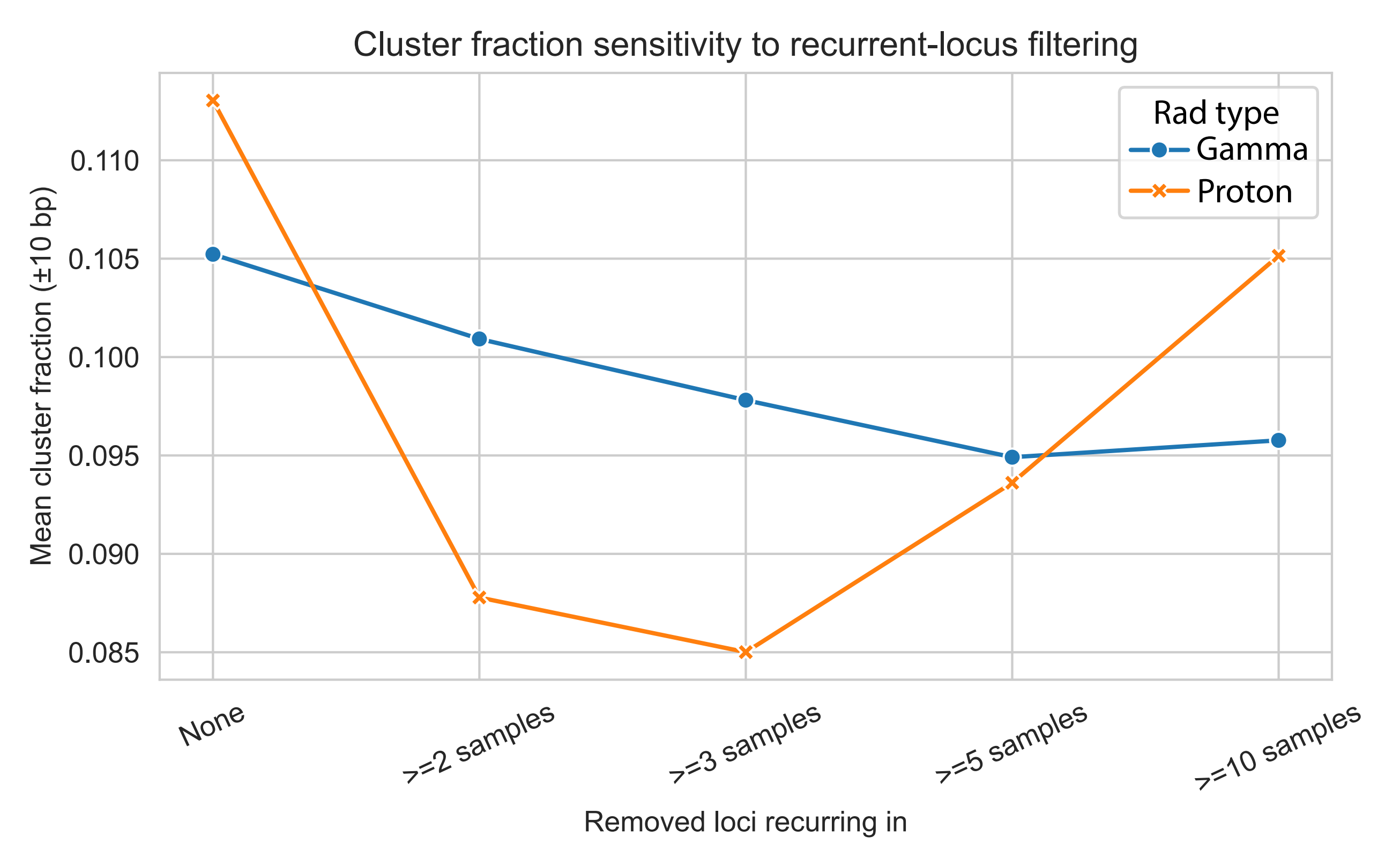
Cluster fraction sensitivity to recurrent-locus filtering
Mean cluster fraction (±10 bp)
0.110
0.105
0.100
0.095
0.090
0.085
Rad type
Gamma
Proton
None
>=2 samples
>=3 samples
>=5 samples
>=10 samples
Removed loci recurring in
