# Supplementary material for: High‐density mutation tracks are associated with proton‐beam irradiation patterns in Sorghum bicolor
Source: Plant Genome. 2026 Jun 29;19(3):e70267. doi: 10.1002/tpg2.70267 (PMC13315512; doi:10.1002/tpg2.70267)
Supplement: Supplementary file 5 [file TPG2-19-e70267-s007.pdf]

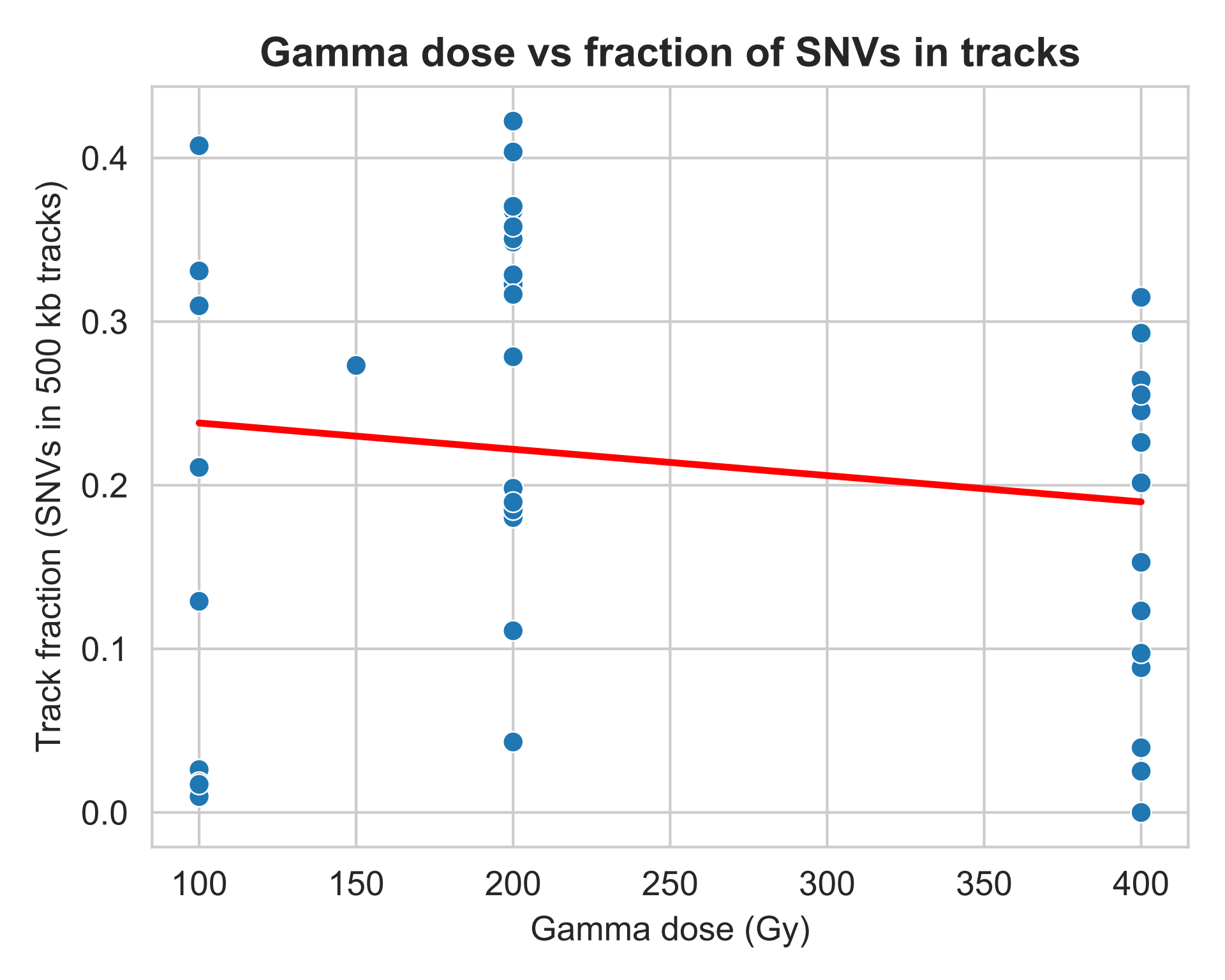
Gamma dose vs fraction of SNVs in tracks
Track fraction (SNVs in 500 kb tracks)
0.0
0.1
0.2
0.3
0.4
100
150
200
250
300
350
400
Gamma dose (Gy)
