# Supplementary material for: High‐density mutation tracks are associated with proton‐beam irradiation patterns in Sorghum bicolor
Source: Plant Genome. 2026 Jun 29;19(3):e70267. doi: 10.1002/tpg2.70267 (PMC13315512; doi:10.1002/tpg2.70267)
Supplement: Supplementary file 6 [file TPG2-19-e70267-s001.docx]

**Supplementary Figures**

**FIGURE S1**

**Callability QC within the GBS-accessible space.** (A) Distribution of callable loci per sample estimated from the SNP matrix panel. (B) Distribution of per-sample missingness within the same panel. Callable-space metrics are comparable between radiation groups, supporting that downstream enrichment and clustering analyses are not driven by gross differences in callability.

**FIGURE S2**

**Feature-specific callable denominators used for callable-space correction.**

Callable loci from the GBS SNP panel were assigned to genomic feature classes using the Rio v2.1 annotation and the repeat-masked intervals. The resulting feature-specific callable fractions (per line) define the expected denominators for enrichment analyses (Supplementary Data S1). This framework explicitly accounts for reduced-representation sampling and enables observed/expected comparisons within the GBS-accessible space rather than the full genome.

**FIGURE S3**

**Sensitivity of short-range clustering to recurrent-locus filtering.**

Short-range clustering was quantified as the fraction of induced SNV events occurring within ±10 bp of another induced SNV on the same chromosome. To test whether apparent clustering could be inflated by a small number of recurrent, highly callable loci, genomic positions were removed if observed in ≥2, ≥3, ≥5, or ≥10 lines, and cluster fractions were recalculated. The cluster fractions changed with recurrent-locus filtering, but clustering signals were not eliminated and were not driven solely by a small set of recurrent loci; summary results are provided in Table S2, and full per-line results are provided in Supplementary Data S1.

**FIGURE S4**

**Dose-independence of mutational clustering in gamma-irradiated lines.**

Scatter plot showing the relationship between absorbed gamma-ray dose (x-axis) and the fraction of total SNVs located within high-density 500-kb mutation tracks (y-axis) for 42 gamma-irradiated sorghum lines. The lack of significant correlation (Spearman’s *ρ* = -0.10, *P* = 0.52) suggests that, within the gamma-treated lines analyzed here, increasing dose was not associated with a higher 500-kb track fraction. This supports the conclusion that the distinct track structures observed in proton-irradiated lines are consistent with differences in spatial clustering between treatment groups in this dataset, rather than a generalized consequence of high mutational load.

**FIGURE S5**

**Correlation between induced mutation density and genomic gene density.**

Scatter plot showing the relationship between normalized gene density and normalized mutation density across 1-Mb genomic windows. Gene density was calculated from the *S. bicolor* reference genome annotation. Gamma-treated windows showed a strong positive correlation with gene density (Pearson *r* = 0.78), whereas proton-treated windows also showed a positive but weaker correlation (Pearson *r* = 0.69). The weaker correlation in proton-treated lines is consistent with localized high-density windows contributing to the mutation-density pattern, in addition to gene-density–correlated callable-site structure.

| **Radiation** | **Feature** | **Observed** | **Expected (callable-corrected)** | **Enrichment (Obs/Exp)** |
| --- | --- | --- | --- | --- |
| Gamma | CDS | 152837 | 144624.5 | 1.056785 |
| Gamma | Promoter | 83942 | 83246.07 | 1.00836 |
| Gamma | Intron (gene body, non-CDS) | 188809 | 181411 | 1.040781 |
| Gamma | Intergenic | 316574 | 332880.4 | 0.951014 |
| Gamma | TE overlap | 184535 | 204831.5 | 0.900911 |
| Proton | CDS | 45443 | 39719.32 | 1.144103 |
| Proton | Promoter | 23848 | 22708.15 | 1.050196 |
| Proton | Intron (gene body, non-CDS) | 54906 | 49656.84 | 1.105709 |
| Proton | Intergenic | 78052 | 90164.7 | 0.86566 |
| Proton | TE overlap | 42216 | 55023 | 0.767243 |

**TABLE S1**

**Callable-space–corrected enrichment of induced SNV events across genomic features.** Observed induced SNV events were assigned to primary genomic feature classes, and TE overlap was evaluated separately. Expected counts were calculated using feature-specific callable fractions estimated from the GBS SNP matrix, thereby correcting for non-uniform sampling of the genome. Enrichment is reported as Obs/Exp within the callable space. TE overlap is not mutually exclusive with CDS, promoter, gene body non-CDS, or intergenic categories. Full statistical outputs, including callable fractions and binomial test *P*-values, are provided in Supplementary Data S1.

| **Radiation** | **Recurrent-locus filter** | **n (lines)** | **Cluster fraction (mean)** | **Cluster fraction (median)** |
| --- | --- | --- | --- | --- |
| Gamma | 0 | 42 | 0.10522 | 0.10619 |
| Gamma | 2 | 42 | 0.10092 | 0.10013 |
| Gamma | 3 | 42 | 0.09781 | 0.09400 |
| Gamma | 5 | 42 | 0.09491 | 0.09286 |
| Gamma | 10 | 42 | 0.09577 | 0.09549 |
| Proton | 0 | 9 | 0.11304 | 0.11392 |
| Proton | 2 | 9 | 0.08778 | 0.09091 |
| Proton | 3 | 9 | 0.08501 | 0.08048 |
| Proton | 5 | 9 | 0.09361 | 0.09397 |
| Proton | 10 | 9 | 0.10514 | 0.10394 |

**TABLE S2**

**Sensitivity of short-range clustering to recurrent-locus filtering.**

Short-range clustering was quantified as the fraction of induced SNV events occurring within ±10 bp of another induced SNV on the same chromosome. To test whether clustering could be inflated by recurrent, highly callable loci, we removed genomic positions observed in ≥2, ≥3, ≥5, or ≥10 lines (recurrent-locus filters) and recomputed cluster fractions by radiation type. Values show the mean and median across lines within each treatment group; the underlying per-line results are provided in Supplementary Data S1.
