# Supplementary figures and images for: High‐density mutation tracks are associated with proton‐beam irradiation patterns in Sorghum bicolor
Source: Plant Genome. 2026 Jun 29;19(3):e70267. doi: 10.1002/tpg2.70267 (PMC13315512; doi:10.1002/tpg2.70267)

Callable-space feature fractions

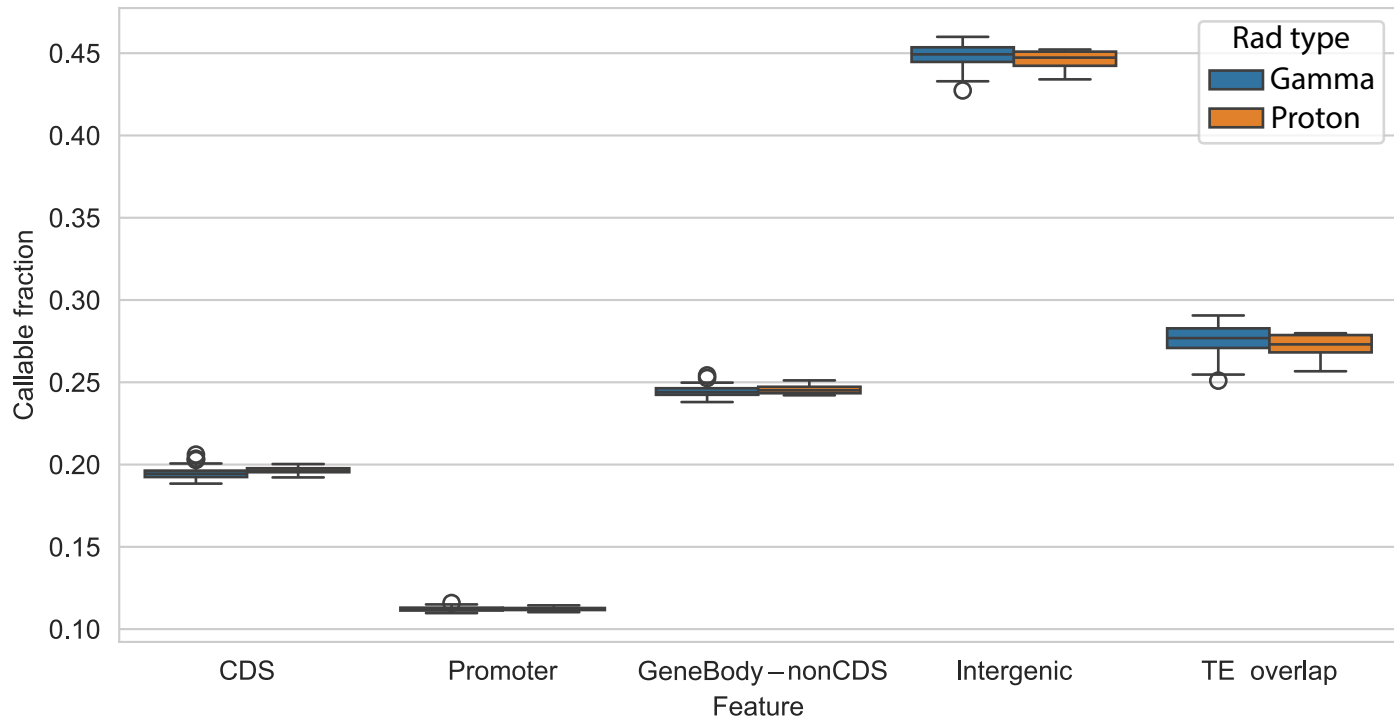

Supplement: Supplementary file 4 — Figure S2. Feature‐specific callable denominators used for callable‐space correction. [file TPG2-19-e70267-s004.pdf]

Cluster fraction sensitivity to recurrent-locus filtering

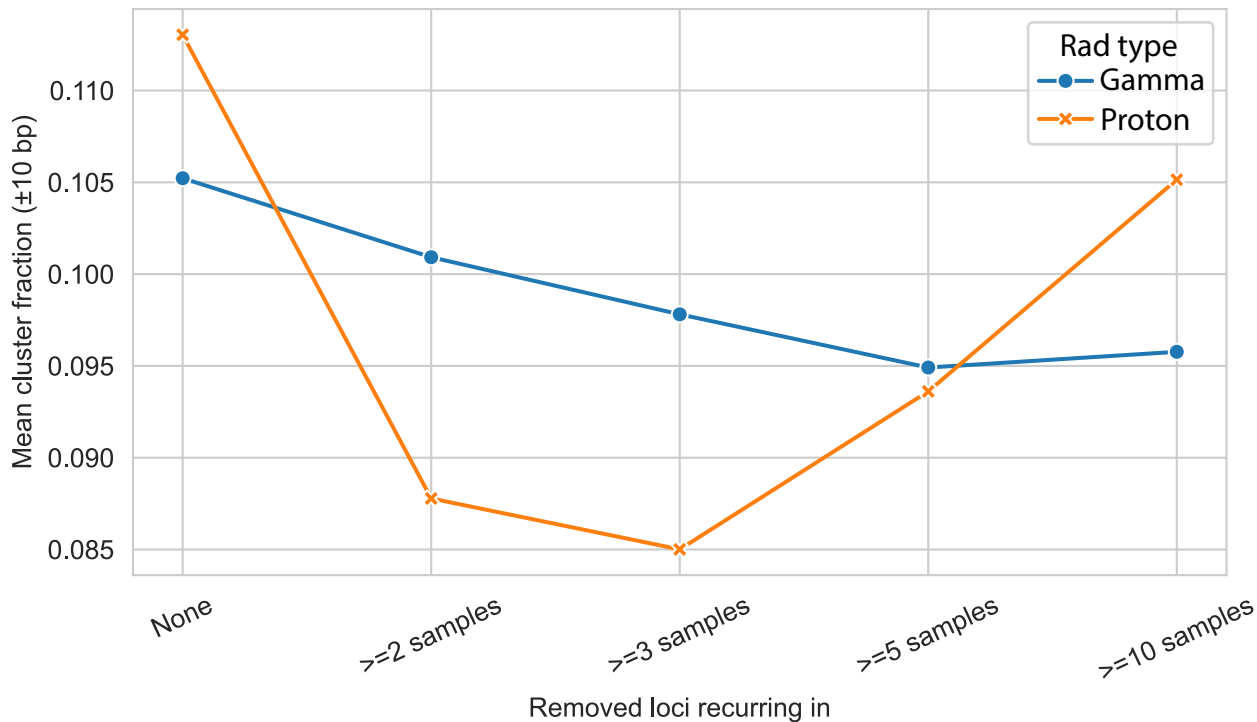

Supplement: Supplementary file 5 — Figure S3. Sensitivity of short‐range clustering to recurrent‐locus filtering. [file TPG2-19-e70267-s006.pdf]

**Gamma dose vs fraction of SNVs in tracks**

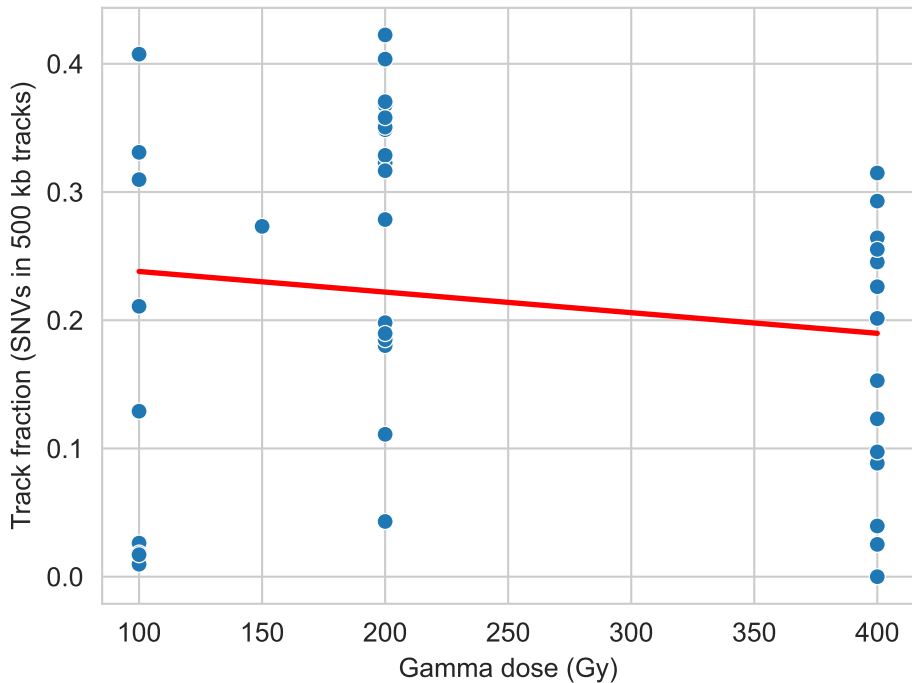

Supplement: Supplementary file 6 — Figure S4. Dose‐independence of mutational clustering in gamma‐irradiated lines. [file TPG2-19-e70267-s007.pdf]

# Mutation Density versus Gene Density

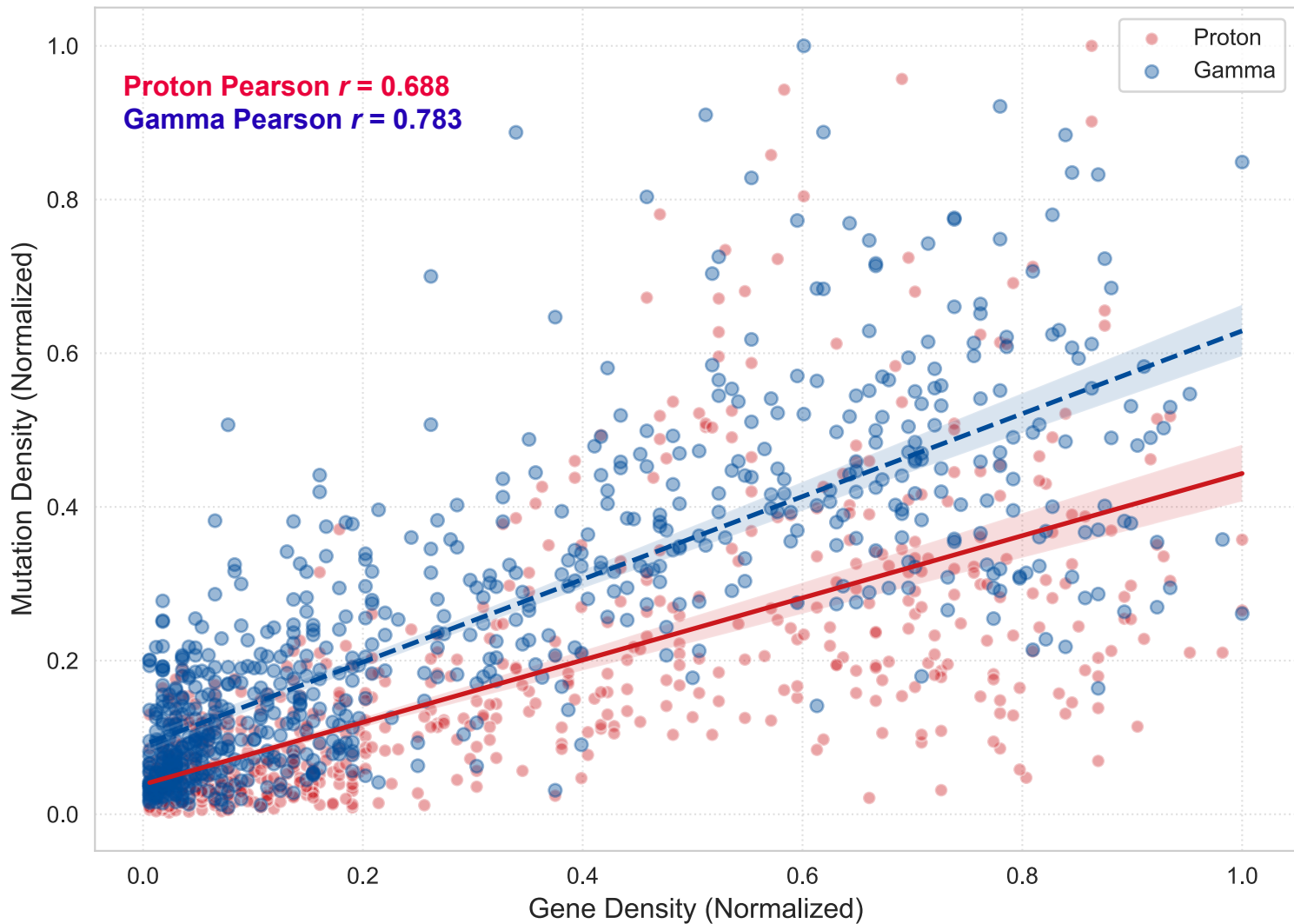

Supplement: Supplementary file 7 — Figure S5. Correlation between induced mutation density and genomic gene density. [file TPG2-19-e70267-s005.pdf]
